# Foot and Ankle Injuries in Elite South African Cricketers: A Descriptive Analysis of Injury Surveillance Data

**DOI:** 10.1007/s43465-023-00934-2

**Published:** 2023-06-26

**Authors:** Benita Olivier, Jaco Naude, Nkazimulo Mnguni, Mmathapelo Thotse, Prudence Phalandwa, Paulo Ferrao, Nikiforos P. Saragas

**Affiliations:** 1 Wits Cricket Research Hub for Science, Medicine and Rehabilitation, Department of Physiotherapy, School of Therapeutic Sciences, Faculty of Health Sciences, University of the Witwatersrand, Johannesburg, South Africa; 2 The Orthopaedic Foot and Ankle Unit, Netcare Linksfield Hospital and the Orthopaedic Department, University of the Witwatersrand, Johannesburg, South Africa; 3 Oxford Institute of Nursing, Midwifery and Allied Health Research, Department of Sport, Health Sciences and Social Work, Oxford Brookes University, Oxford, UK

**Keywords:** Cricket injuries, Bowlers, Batters, Fielders, Lower limb injury, Epidemiology

## Abstract

**Introduction:**

Injury surveillance is an important part of injury risk reduction in the sporting population. This study describes the type, side (dominant or non-dominant), occurrence, impact, activity of onset, and severity of foot and ankle injuries in elite South African male and female cricketers.

**Methods:**

Foot and ankle injuries sustained by elite cricket players between 2018 and 2021, obtained from the records of Cricket South Africa, were descriptively analysed.

**Results:**

A total of 104 foot and ankle injuries in 82 players were recorded. The majority (*n* = 100; 96%) of injuries were on the non-dominant side. Bowling (*n* = 31; 30%) and fielding (*n* = 20; 19%) contributed to most injuries. The majority were first-time (*n* = 83; 80%) and non-impact injuries (*n* = 62; 60%). Fifty percent (*n* = 52) of injuries rendered players unable to participate in at least one match or practice session. Lateral ankle ligament injury was the most common injury sustained (*n* = 36; 35%).

**Conclusion:**

The findings from this study can inform future researchers and assist healthcare service needs relating to injury risk reduction and management programmes. Effective rehabilitation programmes may reduce the risk of reinjury. Ideally, these programmes need to be role specific.

## Introduction

Cricket is a popular sport played globally. Cricket is played over three formats, namely Test cricket (1–5 days), One-day (50 overs), and T-20 cricket (20 overs). Limited-overs formats (20 and 50 overs) are frequently played at a greater intensity than the longer format of the game. Evidence shows that the addition of a shorter, more intense T-20 format in the last decade and a half has resulted in injury spikes due to increased intensity and workload [1]. In addition to workload, other factors such as shoulder range of movement, dynamic lower limb balance and lumbar proprioception have been shown to contribute to the risk of injury [2].

Cricket is considered a non-contact sport with a relatively low injury risk compared to other sports. However, impact and gradual onset injuries are still prevalent. Impact injuries result from contact with the ball, ground, or another player [3]. Gradual onset injuries are non-impact injuries that result from a compounding effect of microtrauma, where repetitive forces result in tissue fatigue over time [3]. Among South African international players, 19% of injuries were of gradual onset [4].

The incidence and type of injuries differs per cricket-specific activity. Bowling accounted for the majority of injuries (41.3%) amongst elite South African cricketers [5], similarly an Australian study [6] reported that most injuries were sustained by bowlers. Fielding typically results in more shoulder injuries [7], whereas bowling mainly causes lumbar and lower limb injuries [8]. The majority of injuries occurred during test matches (43%), and 20% occurred during practices [4].

The lower limb is the most injured body area in cricket. Lower limb injuries which include the hamstring, knee, quadriceps, shin, foot, and ankle, account for 49.8% of all injuries in South African cricket players [5]. In England, 45% of injuries are lower limb injuries [9]. Foot and ankle sprains leading to match time-loss had a prevalence between 0.8% [10] and 1.4% [6]. Elite Australian fast bowlers sustained the majority (*n* = 34) of the 53 match time-loss injuries over three seasons when compared to batters (*n* = 14) and spinners (*n* = 5) [10]. In another study, the medical attention injury definition was applied [3] which included both time-loss and non-time-loss injuries and determined that the prevalence of ankle and foot injuries was 6.8% and 4%, respectively [11].

In cricket, the severity of an injury is determined by how much match time is lost. The updated consensus on injuries in cricket describes a significant injury or medical condition as one that prevents a player from being available for selection or prevents a player from being able to bat, bowl or be a wicketkeeper during a match [3]. In one study, players were unable to participate in training or matches due to an ankle injury for a total of 154 days over 25 months, which amounted to an average of 19.3 days per injury [11]. Injuries not only prevent players from taking part in a match or tournament but may also alter player performance levels after recovery.

Injury surveillance gives insight into the type, nature, activity of onset, and severity of the injury which is important in contributing to injury risk reduction efforts and healthcare services. There are currently no studies describing foot and ankle injuries in the elite South African cricketer. This study aims to describe the type, side (dominant or non-dominant), occurrence, impact, activity of onset, and severity of foot and ankle injuries in male and female elite South African cricket players. The results from this study will provide more insight into foot and ankle injuries, and the findings will aid future research in injury risk reduction and management.

## Materials and Methods

### Study Design and Setting

This study is a retrospective record review.

### Study Participants

Clinical records of all male and female elite South African cricket players, contracted by Cricket South Africa, who sustained a foot or ankle injury between 2018 and 2021 were reviewed.

The definition of injury is that of “medical attention injuries”, which includes both time-loss and non-time-loss injuries and is defined as “any health-related condition that required medical (or medical staff) attention and had the potential to affect cricket training or playing” [3, p2].

### Procedures

Cricket South Africa’s team physiotherapists or sports physicians recorded all injuries sustained by elite cricket players (contracted Cricket South Africa) on the Cricket Clinic software (MicroZone Solutions). Cricket South Africa granted permission to conduct this study, and ethical clearance was obtained from the associated tertiary institution. Clinical records were assigned numerical identification numbers, and injury-specific data was extracted from the clinical records. As this was a retrospective record review, formal consent from players was not required. The following data were extracted: gender, player role (batting/bowling/fielding) injury diagnosis (type of injury), date of injury, date when the player was cleared to play (to calculate return to play), activity of onset (the activity which resulted in injury), impact or non-impact mechanism, dominant or non-dominant side, first-time or recurring (occurrence) and injury severity (time-loss or non-time-loss). Injuries were categorised by an orthopaedic surgeon with a special interest in foot and ankle conditions into the following: Achilles tendonitis, heel bruising, lateral ankle ligament injury, fractures of the foot and ankle, posterior ankle impingement, plantar fasciitis, medial ankle ligament injury, foot laceration, peroneal tendinitis, syndesmosis injury, and sesamoiditis. The remaining injuries were classified as “unable to categorise” due to lack of detail in the online records.

### Data Reduction and Analysis

The data were captured and examined for completeness, where after it was imported into IBM SPSS Statistics (version 27.0, Armonk, NY: IBM Corp). Descriptive analysis was performed and data were presented as frequency, percentages, mean, standard deviation, median or interquartile range (IQR). The recovery period of foot and ankle injuries for each year was calculated using the date difference between the date of injury and the date cleared to play. The number of days to return to play is presented as median and interquartile range (IQR).

## Results

### Participants and Number of Foot and Ankle Injuries Sustained

The study presents the data of 82 elite South African cricket players who sustained 104 foot and ankle injuries from 2018 to 2021. Of the 82 players, 6 were female, and 76 were male. The six female players sustained 1 injury each, while the 76 male players sustained 98 injuries in total. Among those who sustained foot and ankle injuries, some players had multiple foot and ankle injuries over the period of 4 years. Sixty-eight players only sustained 1 foot or ankle injury each, 11 players sustained 2, 2 players sustained 3, and 2 players sustained 4 foot or ankle injuries each.

The number of foot and ankle injuries sustained each year is presented in Table 1 and includes 30 (28.8%) foot and 74 (71.2%) ankle injuries.

**Table 1:** Number of foot and ankle injuries sustained each year (*n* = 104)

Year	Foot injuries *n* (%)	Ankle injuries *n* (%)	Total *n* (%)
2018	5 (23.8)	16 (76.2)	21 (20.2)
2019	9 (28.1)	23 (71.9)	32 (30.8)
2020	8 (36.4)	14 (63.6)	22 (21.2)
2021	8 (27.6)	21 (72.4)	29 (27.9)

### Description of Foot and Ankle Injuries

Four foot and ankle injuries (*n* = 4; 4%) were on the dominant side, while all other foot and ankle injuries were sustained on the non-dominant side (*n* = 100; 96%) (Table 2). Most injuries occurred for the first time (*n* = 83; 80%) and were non-impact injuries (*n* = 62; 60%). Fifty percent (*n* = 52) of injuries rendered players unable to participate in at least one match or practice session.

**Table 2:** Foot and ankle injuries in terms of dominance, occurrence, impact, and injury significance (*n* = 104)

	*n* (%)
Dominance	
Dominant side	4 (4)
Non-dominant side	100 (96)
Occurrence	
First-time injury	83 (80%)
Recurring injury	16 (15%)
Chronic injury	5 (5%)
Impact vs non-impact	
Impact	42 (40%)
Non-impact	62 (60%)
Injury significance	
Unable to participate in match and/or practice	52 (50%)
Limited participation in training or match	32 (31%)
Rested but eligible for full participation in a match	19 (18%)
Not completed	1 (1%)

### Activities of Onset

**Fig. 1:**
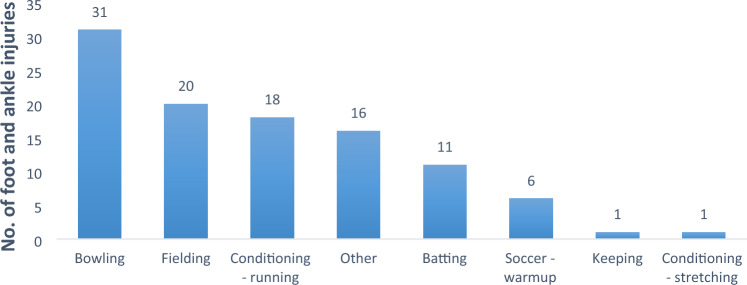
Activities onset of foot and ankle injuries (*n* = 104)

Most foot and ankle injuries occurred during bowling (*n* = 31). Figure 1 shows the activities that the players engaged in at the time of injury.

### Type of Injuries and Activity of Onset

Lateral ankle ligament injury was the most common injury sustained by cricketers (*n* = 36; 35%), with fielding being the most common activity in which the injury was sustained (*n* = 12; 33%). Table 3 shows the injury diagnoses and the activity of onset (type of activity at time of injury).

**Table 3:** Foot and ankle injury diagnoses in terms of activity of onset

	Total injuries *n* (%)^a^	Injuries sustained during bowling *n* (%)^b^	Injuries sustained during batting *n* (%)^b^	Injuries sustained by fielding *n* (%)^b^	Injuries sustained by conditioning—running *n* (%)^b^	Injuries sustained by soccer—warmup *n* (%)^b^	Injuries sustained by keeping *n* (%)^b^	Injuries sustained by conditioning—stretching *n* (%)^b^	Injuries sustained by other *n* (%)^b^
Lateral ankle ligament injury	36 (35)	07 (19)	1 (3)	12 (33)	7 (19)	3 (8)	0 (0)	1 (3)	5 (14)
Medial ankle ligament injury	04 (4)	02 (50)	0 (0)	01(25)	0 (0)	1 (25)	0 (0)	0 (0)	0 (0)
Fractures foot and ankle	05 (5)	1 (20)	03 (60)	0 (0)	0 (0)	0 (0)	0 (0)	0 (0)	1 (20)
Posterior ankle impingement	07 (8)	05 (71)	0 (0)	1 (14)	0 (0)	0 (0)	0 (0)	0 (0)	1 (14)
Heel bruising	03 (3)	02 (67)	0 (0)	0 (0)	1 (33)	0 (0)	0 (0)	0 (0)	0 (0)
Achilles tendonitis	07 (7)	02 (29)	1 (14)	1 (14)	2 (29)	0 (0)	0 (0)	0 (0)	1 (14)
Peroneal tendonitis	07 (7)	01 (14)	1 (14)	2 (29)	3 (43)	0 (0)	0 (0)	0 (0)	0 (0)
Plantar fasciitis	03 (3)	1(33)	0 (0)	0 (0)	2 (67)	0 (0)	0 (0)	0 (0)	0 (0)
Foot laceration	04 (4)	02 (50)	0 (0)	0 (0)	0 (0)	0 (0)	0 (0)	0 (0)	02 (50)
Syndesmosis injury	1 (1)	0 (0)	0 (0)	1(100)	0 (0)	0 (0)	0 (0)	0 (0)	0 (0)
Sesamoiditis	02 (2)	0 (0)	1 (50)	0 (0)	0 (0)	0 (0)	0 (0)	0 (0)	1 (50)
Unable to categorise	25 (24)	08 (32)	04 (16)	02 (08)	03 (12)	02 (08)	01 (04)	0 (0)	05 (20)

^a^Percentage calculated as: no of injuries of this type/total number of injuries, i.e. 104

^b^Percentage calculated as (using the example of ankle ligament injuries in bowlers): no of ankle ligament injuries sustained by bowlers/total no of ankle ligament injuries

### Recovery Timeframe for Foot and Ankle Injuries

Table 4 presents the timeframe for foot and ankle injuries to resolve in terms of the median number of days from the date of injury until the date the player was cleared to play. On average players took longer to return to play after an ankle injury as compared to a foot injury. Data from 2021 are not presented in this table since there were still foot and ankle injuries that were unresolved in 2021.

**Table 4:** Recovery timeframe of foot and ankle injuries

Year	Foot injuries Number of days median (IQR)	Ankle injuries Number of days median (IQR)
2018	7 (11.5)	97 (121)
2019	23.5 (50.5)	18.5 (36.5)
2020	25 (39)	55 (108)
Total	14 (40)	34 (88)

*IQR* interquartile range

### Injuries to Other Body Areas

**Fig. 2:**
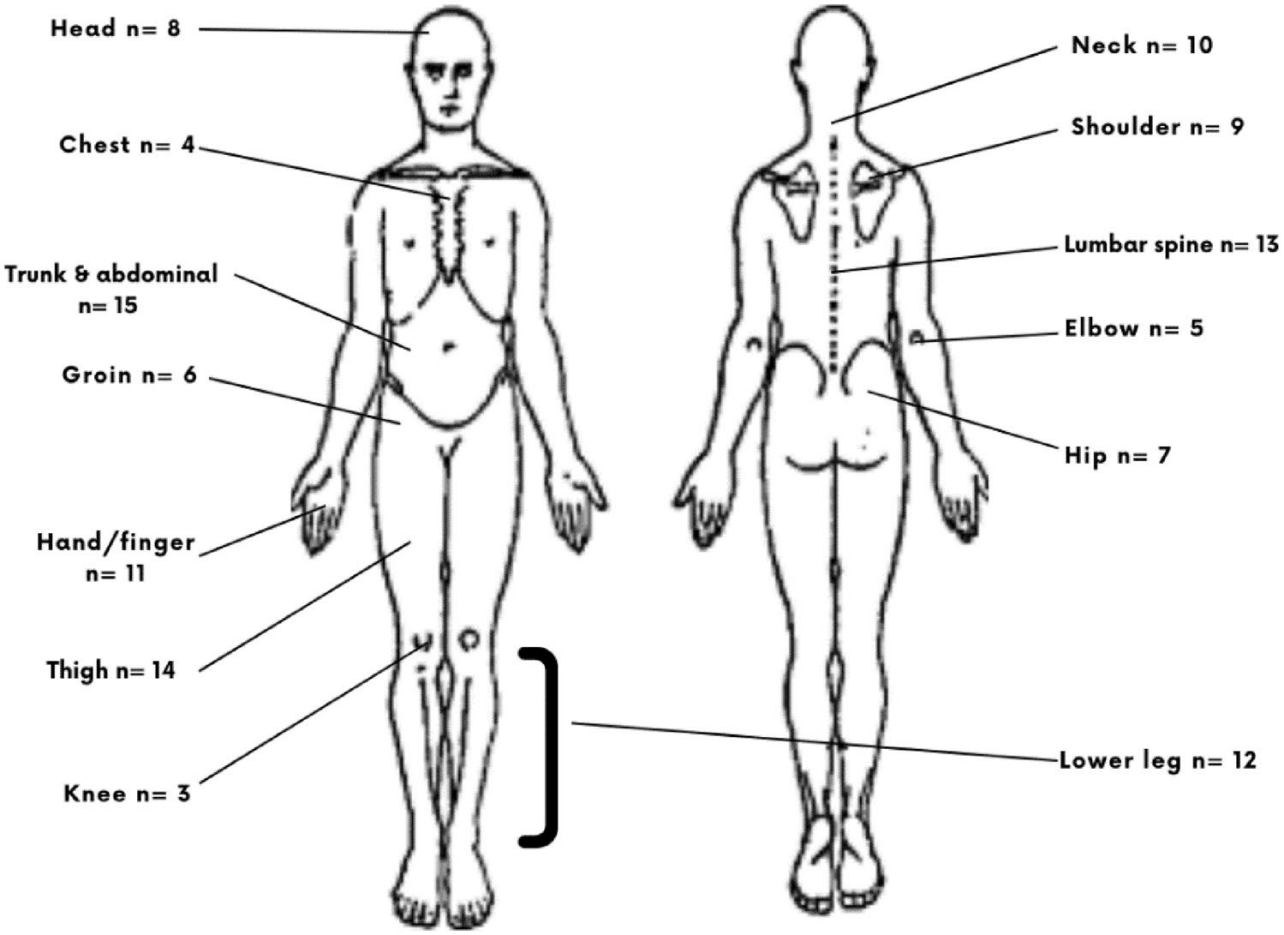
Other injuries sustained by the players, along with the foot and ankle injuries

Cricket players who sustained foot and ankle injuries also sustained injuries to other body areas. The injured areas included the back (*n* = 23), lower leg (*n* = 16), knee (*n* = 19), thigh (*n* = 64), head (*n* = 8), neck (*n* = 3), hip and groin (*n* = 40), shoulder (*n* = 27), elbow (*n* = 6), hand/fingers (*n* = 39), trunk and abdomen (*n* = 17), lumbar spine (*n* = 15), pelvis and buttocks (*n* = 7) and some unspecified areas (Fig. 2).

## Discussion

### Participants and Number of Foot and Ankle Injuries Sustained

This study set out to describe the foot and ankle injuries sustained by male and female elite South African cricket players. This is the first study to focus a specific body area namely the foot and ankle to describe the type, side, occurrence, impact, activity of onset and severity in elite South African cricketers. Results can be used to aid future research in the aetiology, prevention and early-identification of foot and ankle injuries. This paper will also be useful to healthcare providers with a special interest in the foot and ankle, especially those involved in cricket.

The elite cricket players in this study sustained 104 injuries over the 4 years study period with more than double the number of ankle injuries as compared to foot injuries. Similar results, i.e. more ankle injuries than foot injuries have also been found in a study by Perera et al. [11]. We expected that the COVID-19 pandemic which reduced play in 2020 and 2021 would have an impact on the number of injuries. The number of injuries in 2020, however, was in line with those sustained in 2018, with more injuries sustained in 2019 and 2021. The data seems to show a trend of amplification of the number of injuries every alternate year and a further exploration over a longer period may reveal if this trend is indeed present.

### Description of Foot and Ankle Injuries

An interesting finding was that 96 of injuries sustained by elite South African cricketers happened on the non-dominant side. Promsri et al. [12] reported that sensorimotor control and specifically postural control may be affected by leg dominance and may increase the risk to injury in well-experienced downhill skiers, while similar studies in cricket are yet to be done.

Another possible explanation for the high number of injuries on the non-dominant foot and ankle was that the asymmetrical bowling action played a role in the findings. Bowling was the most frequent activity in which foot and ankle injuries were sustained. During the bowling action, the front foot experiences high forces and, as the stride length increases, the amount of force increases as well [13]. The front foot is contra-lateral to the bowling arm, which partially rationalises the high number of foot and ankle injuries on the non-dominant side. A common foot and ankle injury amongst bowlers is posterior ankle impingement [14] due to the plantar flexed position during the first 65% of stance phase and specifically at peak plantarflexion just after front foot contact [13]. In our study, 5 out of the 31 injuries experienced during bowling was that of posterior ankle impingement. This was the second highest injury diagnoses after lateral ligament sprains. Ankle and foot sprains are one of the injury types that were most commonly associated with high bowling workloads in fast bowlers [8]. In our study, it is unclear if the upper vs lower body dominance differed in some players. Also, while approximately 24% of batters [15] and 8% of fast bowlers [16] are left-handed as determined by other studies, many bowlers and batters, for example, bowl right but bat left. Future studies need to consider the role of this in their analysis.

Considering that 60 of the injuries sustained were of a non-impact nature, workload, biomechanical and neuromuscular risk factors may have played a role in the cause of these injuries [2]. Although this study cannot determine cause and effect due to its nature, its findings can offer guidance on the importance of the non-dominant side in prevention, prehabilitation and rehabilitation. Future research can give more insight into this matter.

The majority (80%) of injuries were first-time injuries which is in line with a study by Orchard et al. [6] where 818 of 886 injuries (92%) over a 10 year period were first-time injuries. One of the biggest risks to reinjury is a previous injury and effective rehabilitation can reduce this risk [17]. Functional instability due to neuromuscular (proprioceptive) deficits predispose athletes to future injury [18], while efficient rehabilitation can limit reinjury. The fact that the majority of foot and ankle injuries occurred for the first time, affords a great opportunity to intervene early and prevent future injury.

### Activities of Onset

Most foot and ankle injuries took place during bowling, followed by fielding. Perera et al. [11] reported that fielding was the activity during which most ankle injuries occurred, while bowling led to the most foot injuries. Bowling involves running, and landing activities [13] while fielding requires the cricketer to run, stop, retrieve and throw the ball [19]. Especially the sliding stop during fielding is associated with some risk of injury [19]. Each activity has its own movement components associated with it and, therefore, its own risk factor profile. Role-specific prevention, prehabilitation and rehabilitation are, therefore, essential.

### Type of Injuries

The most common foot and ankle injury sustained by elite South African cricketers was a lateral ankle ligament injury, which was mostly sustained during fielding. In the study by Leary and White [9], contusions/haematomas (41%) were the most commonly encountered foot and ankle injury followed by ligament/joint sprains (29%). The anatomy and biomechanics of the ankle render itself more susceptible to lateral ligament injuries. The anterior talofibular ligament is the lateral ankle ligament which is most commonly injured and displays lower maximal load and energy to failure under tensile stress as compared to the other lateral ankle ligaments. There are numerous factors that can predispose an individual to lateral ligament injury, such as increased genu varum, hindfoot varus, ligamentous laxity, poor postural control and reduced proprioception [20].

Much research has been done into prevention programmes for lateral ankle ligament injuries. Knowing that lateral ankle ligament injury is also the most prevalent foot and ankle condition amongst cricketers can encourage cricket-specific research in this topic area.

### Recovery Timeframe for Foot and Ankle Injuries

Half the foot and ankle injuries sustained lead to players missing at least one match or practice session. Ankle injuries took longer to resolve and required more days out of play than foot injuries. One study found that the average number of days those players missed due to an ankle injury was 19.3 days [11]. Missed playing time is an important aspect considering not only the impact on the team and its performance but also the impact on the quality of life of the player due to the inability to accomplish functional activities of daily living.

### Injuries to Other Body Areas

Many of the players who sustained foot and ankle injuries, also sustained injuries to other body areas. As a result, the authors feel that this is an important finding as the foot and ankle play an important role in the biomechanics or kinetic chain of the rest of the body. Future research need to explore the link between injury rates in different body areas.

### Limitations

Some injury diagnoses on the Cricket Clinic Database did not contain enough detail to be categorised into a specific diagnosis. These injuries were labelled as “unable to categorise”. Inaccurate or missing data are typical challenges when working with big databases. Training and emphasising the value of injury surveillance systems will contribute to more accurate reporting [21]. Due to its study design, this study cannot determine causality. Prevalence and incidence of injury could not be calculated, as information on uninjured players was not available.

## Conclusion

The majority of foot and ankle injuries occur on the non-dominant side were first-time injuries and of a non-impact nature. Most foot and ankle injuries occurred during bowling. Lateral ankle ligament injury was the most common injury sustained, which mostly occurred during fielding. Half of all foot and ankle injuries lead to loss of match or practice time. Effective rehabilitation programmes may be able to reduce risk of reinjury, ideally these programmes need to be role specific. Efforts to improve the accuracy of data in large injury surveillance systems are essential.

## Data Availability

Data cannot be made available due to ethical and legal governance structures in place.
